# Supplementary material for: In situ novel environment assay reveals acoustic exploration as a repeatable behavioral response in migratory bats
Source: Sci Rep. 2021 Apr 14;11:8174. doi: 10.1038/s41598-021-87588-y (PMC8046999; doi:10.1038/s41598-021-87588-y)
Supplement: Supplementary file 1 — Supplementary Information 1. [file 41598_2021_87588_MOESM1_ESM.pdf]

Supplementary Information

In situ novel environment assay reveals acoustic exploration as a repeatable behavioral response in migratory bats

Theresa Schabacker, Oliver Lindecke, Sofia Rizzi, Lara Marggraf, Gunārs Pētersons,
Christian C. Voigt, Lysanne Snijders

Supplementary Tables

Supplementary Table 1. Descriptive statistics of response measures.

Category	Response measure	Min	1st Q	Median	Mean	3rd Q	Max	N/A
Emergence	Body emergence latency (s)	0.91	16.56	48.96	51.09	79.96	161.76	20
	Head emergence latency (s)	0.18	11.32	33.36	41.75	68.53	146.75	16
	Emergence duration (s)	0.56	3.16	4.92	10.61	10.14	114.12	20
Spatial	Number of chambers visited	1	3	9	9	12	25	20
	Number of unique chambers visited	1	3	6	5.25	7	9	20
Acoustic	Number of calls after body emergence	170	946	1227	1177	1482	2127	20
	Number of calls after head emergence	238	994	1274	1250	1574	2342	20
	Number of calls during emergence	4	24	56	72.81	100.5	335	20
Other	Number of head pokes	0	0	1	1.44	2	7	20
	Number of air puffs	0	3	5	5.75	8	22	20
	Upside down crawling	Yes (n = 23)			No (n = 68)			20

Supplementary Table 2. Spearman correlations between response measures within the same behavioral category.

Category	Response measure	Response measure	r_s	p
Emergence	Body emergence latency (s)	Head emergence latency (s)	0.93	< 0.001
	Emergence duration (s)	Head emergence latency (s)	0.25	0.017
	Emergence duration (s)	Body emergence latency (s)	0.51	< 0.001
Spatial	Number of chambers visited	Number of unique chambers visited	0.89	< 0.001
Acoustic	Number of calls after body emergence	Number of calls after head emergence	0.98	< 0.001
	Number of calls during emergence	Number of calls after head emergence	0.34	0.001
	Number of calls during emergence	Number of calls after body emergence	0.22	0.04

Supplementary Table 3. Linear mixed model summary using Type II Wald Chi-square tests for effects of independent variables on 'Acoustic exploration' (residuals of the number of echolocation calls as function of the number of chambers visited). Individual ID was added as random effect. Continuous variables were scaled. Significant effects are reported in bold. Number of observations = 85.

	Estimate	SE	χ^2	df	p
**Test (Second)**	**-344.60**	**52.43**	**43.20**	**1**	**< 0.001**
**Sex (Male)**	**144.71**	**69.44**	**4.34**	**1**	**0.04**
Weight*	5.38	60.04	0.008	1	0.93
Forearm	22.30	38.74	0.33	1	0.56
**Direction (North-South)**	**-93.99**	**43.81**	**4.60**	**1**	**0.03**
**Direction (East-West)**	**85.95**	**42.36**	**4.12**	**1**	**0.04**
Time since sunset*	-55.88	28.70	3.79	1	0.052
Supplemental food (Yes)*	-113.33	71.10	2.54	1	0.11
Orientation test (Before)*	28.35	133.61	0.05	1	0.83

**VIF > 2.5, interpret results with caution*

Supplementary Table 4. Generalized linear mixed model summary using Type II Wald Chi-square tests for effects of independent variables on the number of chambers visited (family = Poisson). Individual ID was added as random effect. In addition, observation number was added to prevent overdispersion. Continuous variables were scaled. Number of observations = 85.

	Estimate	SE	χ^2	df	p
Test (Second)	-0.28	0.14	3.75	1	0.053
Sex (Male)	0.04	0.22	0.03	1	0.86
Weight*	-0.07	0.14	0.26	1	0.61
Forearm	-0.03	0.11	0.11	1	0.74
Direction (North-South)	-0.03	0.12	0.08	1	0.77
Direction (East-West)	0.01	0.12	0.01	1	0.91
Time since sunset*	0.05	0.08	0.41	1	0.52
Supplemental food (Yes)*	-0.24	0.18	1.81	1	0.18
Orientation test (Before)*	0.29	0.36	0.64	1	0.42

**VIF > 2.5, interpret results with caution*

Supplementary Table 5. Linear mixed model summary using Type II Wald Chi-square tests for effects of independent variables on the body emergence latency (log-transformed). Individual ID was added as random effect. Continuous variables were scaled. Significant effects are reported in bold. Number of observations = 104.

	Estimate	SE	χ^2	df	p
Test (Second)	-0.34	0.23	2.31	1	0.13
Sex (Male)	-0.003	0.31	0.0001	1	0.99
**Weight***	**0.38**	**0.19**	**4.05**	**1**	**0.04**
Forearm	0.07	0.15	0.23	1	0.63
Direction (North-South)	0.23	0.16	2.22	1	0.14
Direction (East-West)	0.07	0.16	0.19	1	0.66
Time since sunset*	0.09	0.10	0.79	1	0.37
**Supplemental food (Yes)***	**1.31**	**0.34**	**14.57**	**1**	**< 0.001**
Orientation test (Before)*	-0.08	0.54	0.02	1	0.88

**VIF > 2.5, interpret results with caution*

Supplementary Table 6. Linear mixed model summary using Type II Wald Chi-square tests for effects of response measures exclusively added as independent variable to the final model for 'Acoustic exploration' (residuals of the number of echolocation calls as function of the number of chambers visited). Individual ID was added as random effect. Significant effects are reported in bold. Number of observations = 85.

	Estimate	SE	χ^2	df	p
Body emergence latency (s, log-transformed)	4.70	26.85	0.03	1	0.86
**Residuals (calls ~ emergence duration)**	**2.36**	**0.57**	**17.18**	**1**	**< 0.001**
**Head pokes in adjacent rooms**	**57.87**	**19.16**	**9.12**	**1**	**0.003**
Number of air puffs	7.71	7.85	0.96	1	0.33
Upside down (Yes)	-128.15	68.94	3.46	1	0.06

Supplementary Figures

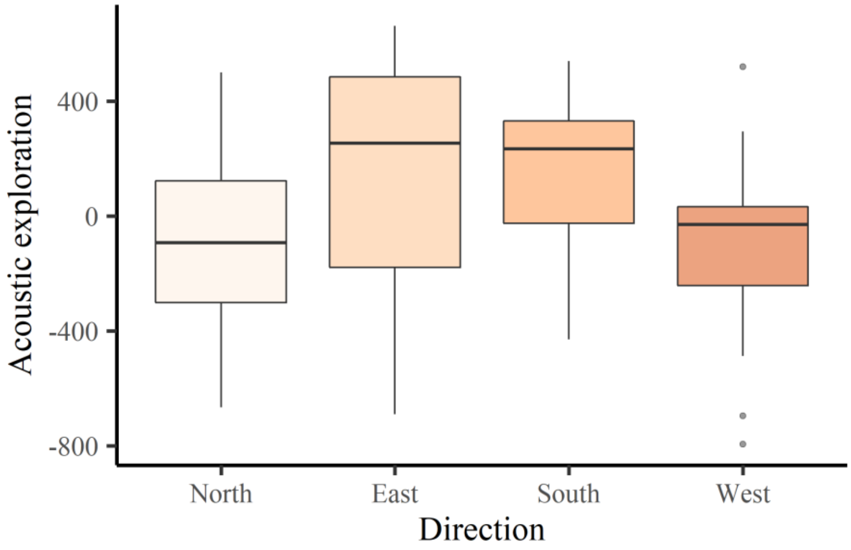

Supplementary Figure 1. Acoustic exploration (number of echolocation calls relative to the number of chambers visited) as function of emergence direction. Note that South-East is the local migratory direction.
